# Measurement of the magnetic moment of single *Magnetospirillum gryphiswaldense* cells by magnetic tweezers

**DOI:** 10.1038/s41598-017-03756-z

**Published:** 2017-06-15

**Authors:** C. Zahn, S. Keller, M. Toro-Nahuelpan, P. Dorscht, W. Gross, M. Laumann, S. Gekle, W. Zimmermann, D. Schüler, H. Kress

**Affiliations:** 1 Biological Physics, Department of Physics, University of Bayreuth, Bayreuth, Germany; 2 Department of Microbiology, University of Bayreuth, Bayreuth, Germany; 3 Department of Molecular Structural Biology, Max Planck Institute of Biochemistry, Planegg-Martinsried, Germany; 4 Theoretical Physics I, Department of Physics, University of Bayreuth, Bayreuth, Germany; 5 Biofluid Simulation and Modeling, Department of Physics, University of Bayreuth, Bayreuth, Germany

## Abstract

*Magnetospirillum gryphiswaldense* is a helix-shaped magnetotactic bacterium that synthesizes iron-oxide nanocrystals, which allow navigation along the geomagnetic field. The bacterium has already been thoroughly investigated at the molecular and cellular levels. However, the fundamental physical property enabling it to perform magnetotaxis, its magnetic moment, remains to be elucidated at the single cell level. We present a method based on magnetic tweezers; in combination with Stokesian dynamics and Boundary Integral Method calculations, this method allows the simultaneous measurement of the magnetic moments of multiple single bacteria. The method is demonstrated by quantifying the distribution of the individual magnetic moments of several hundred cells of *M. gryphiswaldense*. In contrast to other techniques for measuring the average magnetic moment of bacterial populations, our method accounts for the size and the helical shape of each individual cell. In addition, we determined the distribution of the saturation magnetic moments of the bacteria from electron microscopy data. Our results are in agreement with the known relative magnetization behavior of the bacteria. Our method can be combined with single cell imaging techniques and thus can address novel questions about the functions of components of the molecular magnetosome biosynthesis machinery and their correlation with the resulting magnetic moment.

## Introduction

**Figure 1:**
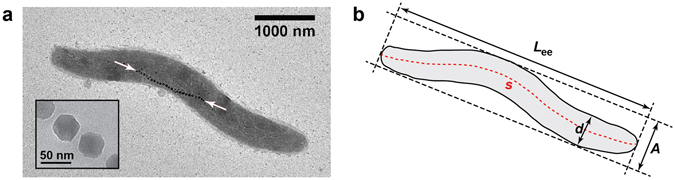
The magnetotactic bacterium *Magnetospirillum gryphiswaldense*. The bacteria possess a helical shape with a length of several micrometers and a diameter of approximately half a micrometer. An intracellular chain of magnetosomes allows them to navigate along magnetic fields. (**a**) Transmission electron microscopy image of a cell with a chain of approximately thirty magnetosomes (white arrows). The inset shows a high resolution image of magnetosomes from another cell. (**b**) We parameterize the helical shape of the cells by the end-to-end length *L*_ee_, the diameter *d*, the arc length *s* and the amplitude *A* of the helix.

The magnetic field of the earth plays a role in the orientation and navigation of a wide variety of organisms including bacteria, algae, bees, pigeons and mice^1^. Magnetic navigation in bacteria was discovered more than 40 years ago^2^. Magnetotactic bacteria, such as the α-proteobacterium *Magnetospirillum gryphiswaldense* ^3^ synthesize magnetosomes, unique intracellular organelles that comprise membrane-enclosed magnetite (Fe_3_O_4_) nanoparticles that allow the cells to align and to navigate along the geomagnetic field^4^. *M. gryphiswaldense* generates cuboctahedron-shaped magnetite crystals with a diameter of approximately 30 to 50 nanometers^5^. The magnetosomes assemble into an intracellular chain, which is positioned at midcell by a dedicated cytoskeletal structure, the actin-like MamK filament^6, 7^. The bacteria are helically shaped with a length of several micrometers and a diameter of approximately half a micrometer (Fig. 1).

*M. gryphiswaldense* has been well investigated previously at the molecular and cellular levels. For example, its motility^8^, its swimming behavior in magnetic fields^9, 10^ and its magnetotaxis and aerotaxis^11^ have been recently investigated. Furthermore, the molecular mechanisms underlying magnetosome biosynthesis and intracellular alignment have been explored thoroughly^4, 5, 7, 12–20^. However, the fundamental physical property enabling *M. gryphiswaldense* to perform magnetotaxis, its magnetic moment, remains to be elucidated at the single cell level. A characterization of the total magnetic moment of a large and unknown number of bacteria as a function of an external magnetic field showed that the remanent magnetic moment has a value of approximately 40% of the saturation magnetic moment and that a magnetic moment of approximately 95% of the saturation magnetization is reached at an external field of approximately 100 mT^21^.

In several studies the average magnetic moment of multiple *M. gryphiswaldense* cells was measured from a bacterial population. In these cases, it was assumed that the cells were identical in size and their helical shape was simplified as a cylindrical or ellipsoidal geometry. These studies yielded average magnetic moments that differ by more than one order of magnitude^10, 22, 23^ despite the fact that the measurements were performed with external magnetic fields below 2 mT, which should lead to comparable magnetic moments that differ only by approximately 2%^21^. In one of these studies, the average magnetic moment was measured using light scattering and the assumption that the bacteria have a cylindrical shape^23^. This approach yielded an average magnetic moment of (25 ± 2) × 10^−16^ A m^2^ at external magnetic fields below 0.9 mT. The length of the model cylinder in this study was determined by a fit to the scattering data and had a value of 1.6 µm which was considered by Reufer *et al*.^10^ to be “unrealistically short”. In their study, Reufer *et al*. determined the average magnetic moment of the bacteria by tracking the motion of nonmotile bacteria in a magnetic field and by assuming that the bacteria had an ellipsoidal shape^10^. This approach yielded a magnetic moment of (2.0 ± 0.6) × 10^−16^ A m^2^ at a magnetic field of 1.5 mT. In a different type of study the bacterial cell dynamics in rotating magnetic fields was measured to determine the ratio of the magnetic moment of the bacteria to their rotational drag coefficient^9^. For this study, it was stated that a magnetic moment of 43 × 10^−16^ A m^2^ would be in agreement with a bacterium that possesses a rotational drag coefficient of an ellipsoid with a long axis of 4 µm and a short axis of 0.5 µm. The large spread of the reported values for the magnetic moment of *M. gryphiswaldense* raises the question of the underlying reasons for these discrepancies. Possible reasons include the approximation of the helical cell shape by ellipsoids and cylinders, the usage of average cell dimensions instead of the individual dimensions of each measured cell or systematic errors in the used measurement techniques.

Besides the abovementioned studies about the magnetic moment of *M. gryphiswaldense*, there are various other characterizations of the magnetic moments of closely related bacterial species such as *M. magnetotacticum* ^24, 25^ and *M. magneticum* ^26^. The latter article addresses the question of potential systematic errors in various measurement techniques and presents a comparison of six different methods to determine the magnetic moments of bacteria. The authors showed that the use of different methods led to magnetic moments that varied by almost one order of magnitude. They found that methods relying on viscous relaxation of the cell orientation gave results that were comparable to magnetosome measurements, whereas methods relying on statistical mechanics assumptions gave systematically lower values. Since living cells were used in the study of Nadkarni *et al*., the authors suggested that the non-thermal noise induced by the living cells is a potential source of error in measurements of the magnetic moment of bacteria.

In summary, the magnetic moments of *M. gryphiswaldense* ensembles have not yet been measured at the single cell level. Furthermore, there are multiple open questions concerning the large discrepancies between the magnetic moments that were reported so far for these and other magnetotactic bacteria: Is it - in the case of *M. gryphiswaldense* - necessary to take the helical shape of the cells into account or is it sufficient to approximate them with a simplified geometry such as a cylinder or an ellipsoid? Is it necessary to take the dimension of each individual cell into account or is it sufficient to use the average dimensions of a bacterial ensemble? Does the use of dead (chemically fixed) cells that do not induce non-thermal noise lead to more consistent results if different methods are compared?

Here we present a method for measuring the magnetic moments of multiple single cells of magnetotactic bacteria simultaneously by analyzing their dynamics in various magnetic fields. We demonstrate the method by quantifying the magnetic moments of more than 350 individual cells of *M. gryphiswaldense*. Inhomogeneous switchable magnetic fields were created using magnetic tweezers (MT). Magnetic tweezers and the comparable technique of magnetic twisting cytometry are versatile biophysical methods for force and torque generation on small length scales and have been applied in single molecule and cellular studies^27–33^. The translational motion of cells was measured in static magnetic field gradients, whereas the rotational motion of cells was measured in alternating magnetic fields. For each bacterium, these measurements yielded the ratio of its magnetic moment to its translational and rotational viscous drag coefficients, respectively.

The translational and rotational viscous drag coefficients were calculated for each bacterium by two methods in the low Reynolds number limit of Stokes flow, both taking into account the helical shape of the bacteria and their individual dimensions. In a Stokesian dynamics approach the bacterial shape was approximated by several thousand small spheres and the hydrodynamic interaction between the spheres was calculated using the Oseen tensor. The second approach was the Boundary Integral Method (BIM) where the bacterium’s surface was discretized as a large set of flat triangles, and no-slip boundary conditions for the flow were used at their surfaces. The flexibility of the two methods allows the application of our approach not only in the case of helically-shaped bacteria but also for the general case of arbitrary cell shapes. In addition to our measurements of the magnetic moments of a large number of bacteria in various external magnetic fields, we also determined the saturation magnetic moment of 50 individual bacterial cells by estimating their magnetosome crystal volume from transmission electron microscopy (TEM) images. Our measurements show that for a typical bacterial population with a length distribution in the range of approximately 2 to 6 µm, the use of mean cell dimensions and the approximation of the cell shape by a simple geometry are sufficient if only population averages of the magnetic moment are needed and if uncertainties on the order of about 10% are acceptable. However this approach leads to an over- or underestimation of up to more than a factor of 2 if it is used to determine the magnetic moment of an individual single bacterium. To determine the magnetic moments of individual single bacteria correctly, their particular shape and size has to be taken into account. Finally, we show that different measurement techniques yield consistent results if dead cells are used, which do not induce non-thermal noise.

## Materials and Methods

### Cell culture and sample preparation

The *M. gryphiswaldense MSR-1* wild type strain was grown under microoxic conditions in 2% oxygen aerated modified flask standard medium (FSM)^34^ containing 50 μM ferric iron citrate. Cultures were incubated at 30 °C with moderate agitation (120 rpm). Exponentially growing cells were fixed in 1.5% formaldehyde for 2 h. Subsequently, 1 ml of culture was centrifuged at 5,000 rpm for 5 min, and the cell pellet was resuspended in a highly viscous solution containing 85% v/v glycerol and 15% v/v water for further analysis.

### Polyacrylamide gel preparation

The polyacrylamide (PAA) substrates were prepared according to a protocol that was published previously^35^, which we adapted for use in our laboratory^36^. Briefly, 40 × 22 mm sized coverslips (Glaswarenfabrik Karl Hecht, Sondheim v. d. Rhön, Germany) were cleaned by sonicating them successively for 10 minutes in 0.2 M EDTA, 10% w/v hydrogen chloride, and 1% v/v 7X-O-Matic (MP Biomedicals Germany, Eschwege, Germany). After each single sonication step, the coverslips were washed in deionized water (DI). The coverslips were air-dried before surface-activation, which was performed to covalently bind the coverslips to PAA. The details of the reaction were described previously^37^. We spread 20 µl 0.1 M sodium hydroxide by rolling a glass Pasteur pipette over each coverslip. When the coverslips were dry, 15 µl (3-aminopropyl)trimethoxysilane (Sigma-Aldrich, St. Louis, MO) was spread and the coverslips were allowed to dry for 5 minutes. The coverslips were washed 3 times in DI and incubated in 200 µl 0.5% glutaraldehyde solution (from 8% stock solution, Sigma-Aldrich) for 30 minutes and washed in DI 3 times again. The coverslips were stored up to 1 month together with desiccant beads (Neolab Migge Laborbedarf-Vertriebs, Heidelberg, Germany). To cover the gels during polymerization and achieve a flat top surface, we coated coverslips with a diameter of 15 mm (Menzel-Gläser, Braunschweig, Germany) hydrophobically with RainX (Krako Car Care International, Altrincham, WA) according to the manufacturer’s protocol to facilitate better detachment of the substrates^38^. To remove dust, the coverslips were cleaned with canned air directly prior to substrate polymerization.

To polymerize PAA substrates, a monomer solution of 5% w/v acrylamide (AA, from 40% w/v stock solution, Sigma-Aldrich) and N,N′methylenebisacrylamide (BIS, from 2% w/v stock solution, Sigma-Aldrich) at a concentration of 0.06% w/v in phosphate-buffered saline (1xPBS, 0.2 g l^−1^ KCl, 8.0 g l^−1^ NaCl, 1.44 g l^−1^ Na_2_HPO_4_, and 0.24 g l^−1^ KH_2_PO_4_ in DI) was prepared. N,N,N′,N′-tetramethylethylenediamine (Thermo Fisher Scientific, Waltham, MA) at a final concentration of 1/2000 v/v was added to catalyze the polymerization reaction. The polymerization reaction was started by the addition of 1/200 v/v freshly prepared 10% w/v aqueous ammonium-persulfate (APS) solution.

To prepare thin substrate layers, 15 µl of the monomer solution was pipetted on a RainX-coated coverslip. A surface-activated coverslip was lowered from the top with the activated side facing downwards until surface tension kept both coverslips in place. This sandwich configuration was suspended on a pair of Pasteur pipettes to polymerize at room temperature and high air humidity of approximately 60–80% to minimize evaporation effects. After polymerization, the round coverslips were removed carefully with forceps and the substrates were washed 3 times in 1xPBS to remove unreacted monomers. The substrates had a thickness that ranged from approximately 30 to 110 µm. Even though the thickness of every substrate varied quite significantly the surface still remained horizontal with inclination angles well below 1°, thus not influencing the measurement. Before the measurement, superparamagnetic beads with a diameter of 4.5 µm (Dynabeads® M-450 Epoxy, Thermo Fisher Scientific) were sedimented on the substrates.

### Magnetic tweezers setup and calibration

The magnetic tweezers (MT) setup is based on an inverted light microscope (Nikon Eclipse Ti-U, Nikon, Tokyo, Japan) with a 20× magnification objective for calibration measurements (CFI Plan Achromat 20× objective, NA 0.4, Nikon) and a 60× magnification objective (CFI Plan Apochromat λ 60× oil objective, NA 1.40, Nikon) for measurements of the bacteria. Image sequences were acquired with a CMOS camera (Orca-flash 4.0 v2, Hamamatsu, Shizuoka, Japan) under bright-field illumination.

The MT consists of a solenoid with a high permeability soft iron core and a power supply with a maximum output power of 10 A (Elektro Automatik, Viersen, Germany). The coil consists of 1420 turns of a copper wire with a diameter of 0.5 mm and the resulting solenoid has a dimeter of 20 mm and a length of 50 mm. The core material is the nickel-iron alloy Mumetall (Vacuumschmelze GmbH, Hanau, Germany), which possesses a magnetic permeability up to µ_max_ = 250000. The core has a cylindrical shape and a conical sharp tip with a core length of 163 ± 2 mm and a tip diameter of 35 ± 2 µm. To increase the magnetic permeability of the Mumetall and therefore the magnetic field that can be generated, the rod was annealed in a magnetic field in hydrogen atmosphere at the Vacuumschmelze Hanau. The coil including the soft iron core can be positioned in *x*-, *y*- and *z*-direction using single-axis translation stages (Mitutoyo, Sakado, Japan and Thorlabs, Newton, USA). The inclination angle of the tip within the sample can be adjusted by a manual rotation stage (Suruga Seiki, Tokyo, Japan). Before and after the usage of the MT the remanent magnetization of the core was reduced by a demagnetizer (Analogis, Falkensee, Germany).

The magnetic forces generated by the MT were calibrated by analyzing the movement of super-paramagnetic particles (Dynabeads M-450) with a diameter of *d* = 4.5 μm within a highly viscous fluid consisting of glycerin and water. We determined the exact concentration of the glycerin in the glycerin-water stock solution by measuring the viscosity of the solution with an Ostwald viscometer. At a temperature of 21.1 °C, we measured a viscosity of *η*_G_(21.1 °C) = 1.23 ± 1.0 Pa *s* which corresponds to a glycerin concentration of 99.79%. For sample preparation, 0.012 ml of the particle-water stock solution was suspended in 1.5 ml of the 99.79% glycerin stock solution. The particle concentration of the resulting solution was 3.2 · 10^6^ particles per ml. For the temperature of T = 22.5 ± 1.0 °C at which the MT calibration measurements were performed, we determined a viscosity of *η*_G_ = 0.95 ± 0.25 Pa *s*. The motion of the particles in the magnetic field was measured by bright-field time-lapse microscopy using the 20× objective and an acquisition rate of 20 frames per second. The particle positions were determined by applying a centroid-based tracking algorithm^39^. The viscous drag force *F*_*d*_ exerted on each particle was calculated by using Stokes law *F*_*d*_ = 3*π* · *η*_G_ · *d* · *v*, where *v* is the velocity of the particle.

### Magnetic tweezers experiments

For the characterization of the magnetic moments of single *M. gryphiswaldense* cells with the MT, fixed bacteria suspended in the 85% (v/v) glycerol solution (dynamic viscosity of 135 mPa s at a temperature of 22.5 °C) were placed on a glass coverslip (No. 1, 18 mm diameter, Marienfeld-Superior, Lauda-Königshofen, Germany) which was mounted into a custom-built aluminum holder. The MT tip was immersed into the sample and the bacterial motion in the magnetic field was monitored with bright-field time-lapse microscopy at room temperature with the 60× objective.

For measuring the translational motion of the bacteria in temporally constant magnetic fields, the current through the MT coil was set to *I* = 0.1 A and the image acquisition rate was 20 frames per second. The motion of the center of mass of each bacterium was tracked manually. Only bacteria with a distance of more than 8 µm from the MT tip surface were tracked to ensure positioning within the well-calibrated area of the MT.

For measuring the rotational motion of the bacteria in temporally varying fields, the magnitude of the current through the coil was set to *I* = 0.008 A, and the direction of the current was alternated periodically. The periodicity of the alternations was sufficiently low to allow all rotating bacteria to finish their motion before the current direction was switched. The image acquisition rate was set to 2 frames per second. For bacteria that were rotating mainly in the image plane, the longitudinal axis of the bacteria was identified manually in each image, and the angle *θ* of the bacterium relative to the magnetic field direction was determined. For bacteria that were rotating mainly perpendicular to the image plane, the time that was need for a full rotation was determined.

### Transmission electron microscopy (TEM)

For conventional TEM analysis, cells were grown at 28 °C under microaerobic conditions, fixed in formaldehyde (1.5%) and concentrated by centrifugation. Next, unstained cells were absorbed on carbon-coated copper mesh grids (Plano, Wetzlar). Bright-field TEM was performed on a FEI CM200 (FEI; Eindhoven, The Netherlands) transmission electron microscope using an accelerating voltage of 160 kV. Images were captured with an Eagle 4 k CCD camera using EMMenu 4.0 (Tietz) and FEI software. Fiji software was used to obtain data regarding the cell dimensions.

### Calculation of the viscous drag coefficients via Stokesian dynamics

The Stokesian dynamics method calculates the flow at zero Reynolds number around an object and its drag by discretizing the surface of the object and by using the flow field of point forces. For details see the Supporting Information and work by Leal^40^.

### Calculation of the viscous drag coefficients by Boundary Integral Method

The Boundary Integral Method solves the Stokes flow at zero Reynolds number by expressing the flow field as integrals over arbitrarily shaped domain boundaries. For details see the Supporting Information and work by Daddi-Moussa-Ider *et al*.^41^ and Guckenberger *et al*.^42^.

## Results

### Magnetic tweezers calibration

**Figure 2:**
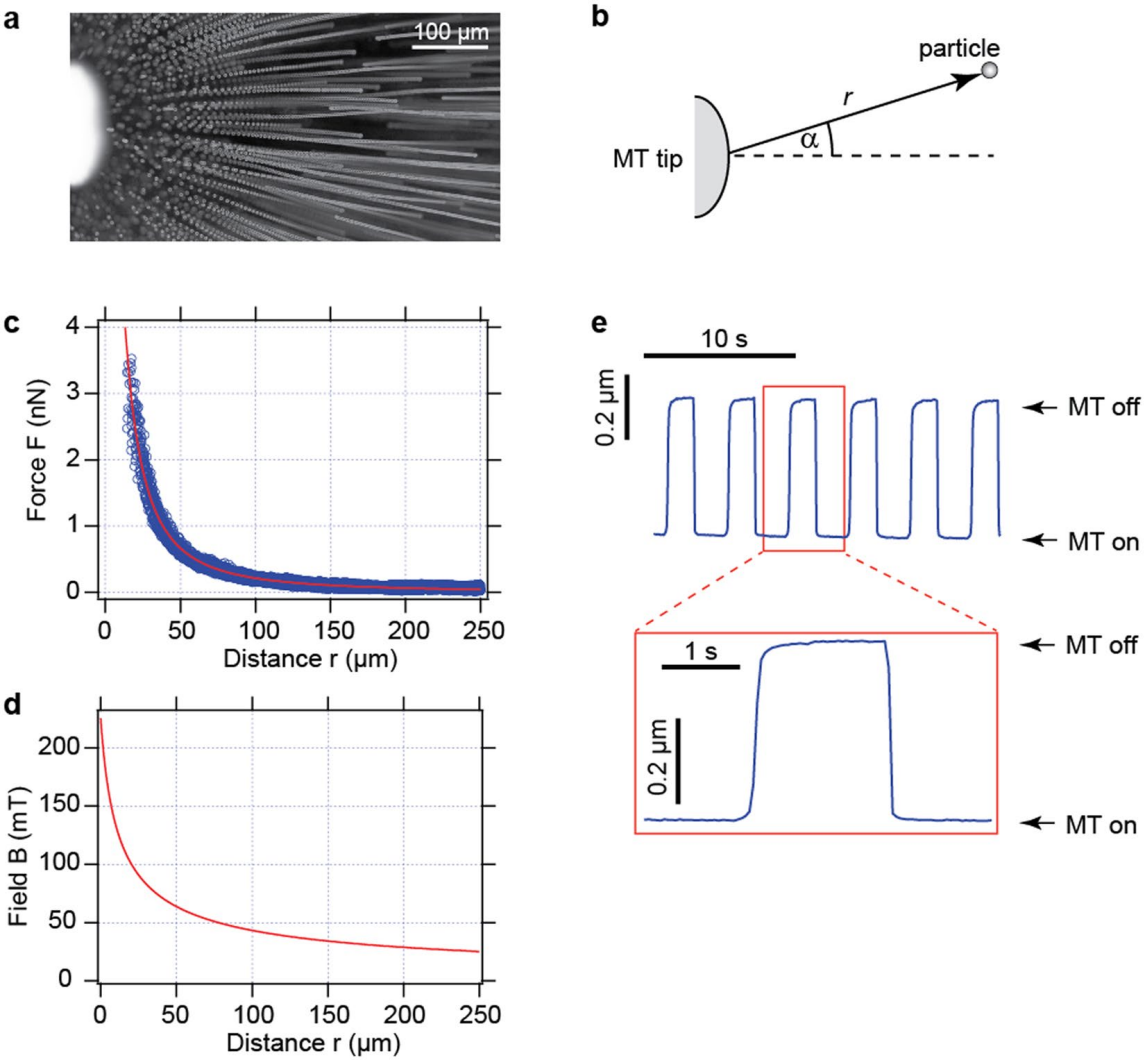
Magnetic tweezers calibration and characterization. The MT were calibrated by tracking the motion of superparamagnetic microparticles towards the MT tip in a highly viscous liquid environment. (**a**) A maximum projection of a time-lapse image sequence with a constant frame rate shows directly the direction of the applied force and the acceleration of the particles towards the tip on the left side. (**b**) The position of a particle is characterized by its distance *r* from the tip and its polar angle *α* with respect to the symmetry axis of the MT. (**c**) The data for the force *F* exerted by the MT on the particles as a function of *r* are shown by the blue circles. The red line shows the fit of Eq. 1 to the data. The current through the coil of the MT was *I* = 0.5 A in this measurement. (**d**) The known magnetization behavior of the superparamagnetic particles allows the calculation of the magnetic field *B* as a function of the distance *r*. (**e**) The field switching time was characterized by tracking the motion of a superparamagnetic particle that was bound to an elastic substrate in a magnetic field that was turned on and off periodically. The data shows the lateral displacement of the bead in the direction of the field. The motion of the particle as a function of time indicated an upper limit for the characteristic times for turning the fields on and off.

The MT system was calibrated using superparamagnetic microparticles with a diameter of 4.5 µm as described in the *Materials and Methods* section. Briefly, the motion of a large number of particles towards the tip of the MT in a highly viscous liquid environment (Fig. 2a) was tracked microscopically using digital image processing. The magnetic forces exerted on the particles as a function of their positions (Fig. 2b) were calculated from the particle velocities and their Stokes drag coefficient (Fig. 2c). At a given electric current *I* through the coil of the MT, the force *F* exerted on a particle depends on the distance *r* between the tip and the particle. The polar angle *α* of the particle position had no detectable influence on the force-distance relationship *F*(*r*) as long as *α* ≤ 40° was fulfilled. The angle *α* = 0° defines the symmetry axis of the MT (Fig. 2b). We therefore limited the tweezers calibration and the subsequent measurements on bacteria to polar angles of *α* ≤ 40° and considered only the distance of the particles and of the bacteria, respectively, for the subsequent data analysis.

The force-distance relationship *F*(*r*) can be described by the equation,

1 $$F(r)=\frac{{F}_{0}}{{[\frac{r}{2{r}_{0{\rm{F}}}}+\frac{1}{2}]}^{{c}_{{\rm{F}}}}}$$

a modified version of the force-distance relation described by Kollmannsberger and Fabry^43^, which has the property that *F*(*r*_0F_) = *F*_0_. For a current of *I* = 0.5 A through the coil of the MT, fitting Eq. 1 to the data yielded *F*_0_ = 5.5 ± 0.1 nN, *r*_0F_ = 9.6 ± 0.2 µm and *c*_F_ = 1.87 ± 0.02 (Fig. 2c).

A particle with a magnetic moment ***µ*** in an inhomogeneous magnetic field ***B*** experiences the force

2 $${\boldsymbol{F}}=({\boldsymbol{\mu }}{\boldsymbol{\nabla }}){\boldsymbol{B}}$$

The known relationship between the magnitude *B* of the external field and the magnitude *µ* of the magnetic moment of the superparamagnetic particles, i.e., the function *µ*(*B*)^44^ allowed us to derive the magnetic field *B* as a function of the distance *r* (Fig. 2d) from the measured relation between the force *F* and the distance *r* (Fig. 2c). We found that the equation

3 $$B(r)=\frac{{B}_{0}}{{[\frac{r}{2{r}_{0B}}+\frac{1}{2}]}^{{c}_{{\rm{B}}}}}$$

is suitable to describe the relationship between the magnetic field and the particle distance. For a current of *I* = 0.5 A through the coil of the MT, the resulting parameters were *B*_0_ = 147 mT, *r*_0B_ = 7.5 µm and *c*_B_ = 0.62. The corresponding field-distance-relation is shown in Fig. 2d. At distances of *r* = 10 … 250 µm, magnetic fields of approximately B = 130 … 25 mT can be achieved. The magnetic H-field of the MT coil scales linearly with the current *I*. However, the magnetic B-field scales less than linearly with the H-field in our parameter range since the permeability of the annealed Mumetall core material decreases with increasing current for H-fields in the range of approximately 0.1 … 10 A cm^−1^ ^45^. With the lowest current (*I* = 0.008 A) that we used, we were able to generate magnetic fields of B = 6 … 55 mT at distances of *r* = 10 … 250 µm.

For experiments in which the magnetic field of the tweezers is abruptly altered (e.g., by changing the direction of the current), it is necessary to know the timescale for changing the field rapidly. To measure an upper limit of this time scale, we placed the tip of the MT close to a superparamagnetic microparticle bound to the surface of an elastic polyacrylamide gel. The MT were turned on and off sequentially and the resulting motion of the particle was tracked microscopically (Fig. 2e). The observed time scale for turning the tweezers on was lower than the time scale for turning them off. An upper limit *τ*_max_ for the tweezers switching time *τ* can be defined as the time after which 95% of the total particle displacement is reached. This definition yields τ_max,on_ = (100 ± 10) ms and τ_max,off_ = (290 ± 50) ms for turning the tweezers on and off, respectively. These time scales represent upper limits for the switching time because they include the finite response time of the gel to a sudden force that is exerted on the gel.

### Rotation of bacteria

Bacteria with a given magnetic moment ***µ*** experience a torque ***T*** = ***μ*** × ***B*** in a magnetic field ***B*** which leads to a rotation of the bacteria if ***µ*** and ***B*** are not exactly parallel or antiparallel to each other. Chemically fixed bacteria (i.e. dead and thus incapable of active swimming) were immersed in a highly viscous glycerol-water mixture (85% v/v glycerol) to slow the rotation to a time scale on the order of ten seconds. This step facilitated tracking of the rotation by time-lapse microscopy with an image acquisition rate of 2 Hz. The bacteria aligned with the field of the MT when the tweezers were turned on for the first time in the sample. After the magnetic field direction was switched by changing the direction of the electric current through the coil, the bacteria rotated 180° to align with the new field direction. Although bacteria that are aligned exactly antiparallel to the magnetic field experience no field-induced torque, thermal fluctuations of the orientation lead to deviations from the instable antiparallel equilibrium orientation. For multiple subsequent measurements, we switched the field with periods that were significantly longer than the time period of a full 180° rotation. These measurements were performed at magnetic field strengths between 6 mT and 23 mT. Measurements in such relatively low magnetic fields yield magnetic moments that are close to the remanent moments of the bacteria. The lower boundary of 6 mT was given by the lowest field strength that we generated in the field of view of the microscope. The upper boundary of 23 mT was given by the highest field strength for which we were able to observe rotation of the bacteria. These magnetic fields were sufficiently low to not alter the direction of the magnetic moments of the bacteria with respect to the bacterial orientation. In contrast, upon application of higher magnetic fields with strengths above 23 mT, we observed that the bacteria did not rotate upon field reversal.

**Figure 3:**
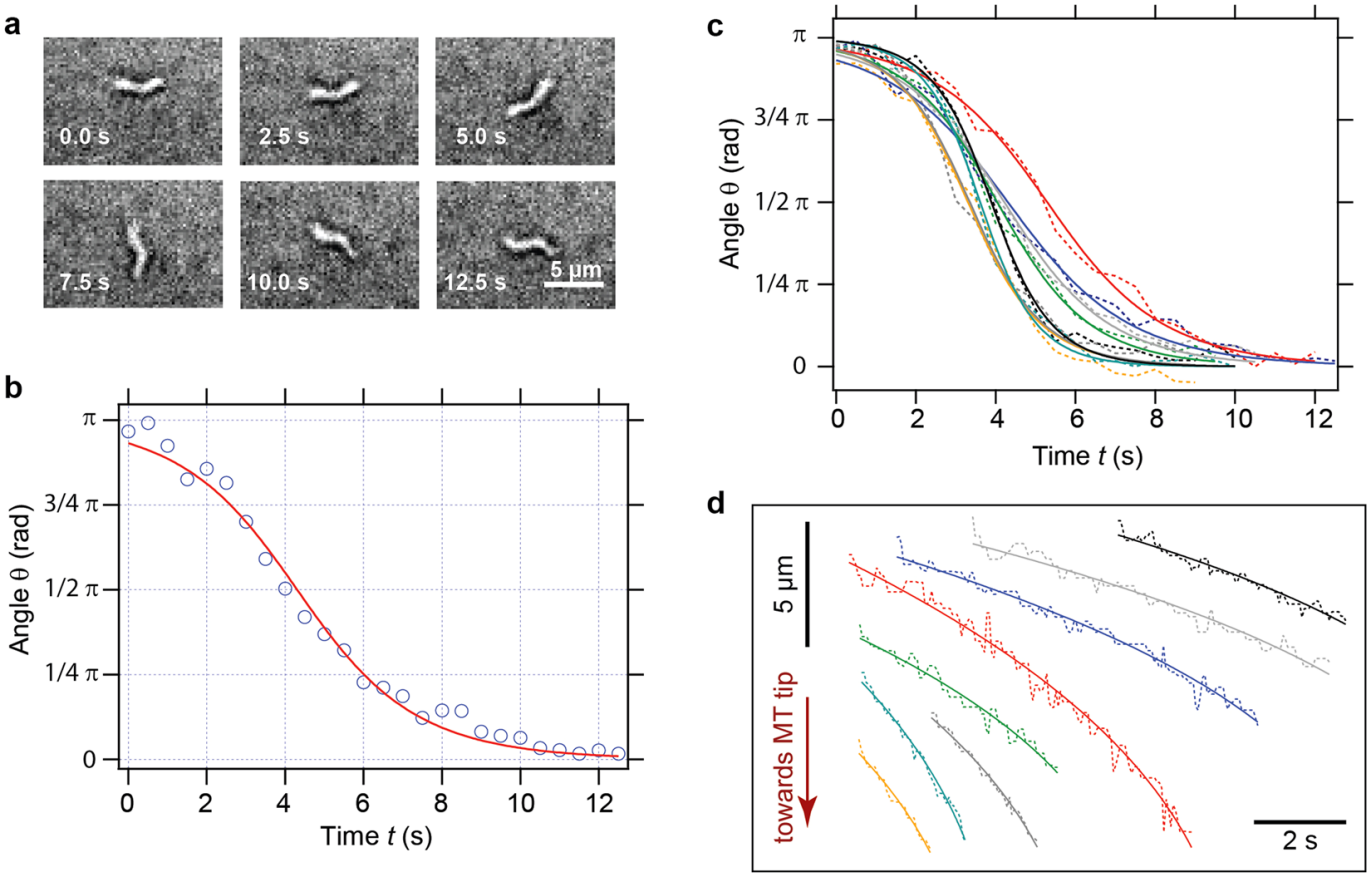
Rotation of *M. gryphiswaldense* in reversed magnetic fields and translation of the bacteria in static magnetic fields. (**a**–**c**) Data for the rotation of bacteria in reversed magnetic fields. (**a**) Frames of a time-lapse microscopy series of a rotating cell. (**b**) Angle ***θ*** of a rotating cell as a function of time t (points) and fit of Eq. 4 to the data (red curve). (**c**) Multiple time series of the rotation angle ***θ*** as a function of time t (dashed curves) and the corresponding fits to Eq. 4 (solid curves). (**d**) Data for the translation of bacteria in static magnetic fields. Multiple time series of the bacterial displacements as a function of time (dashed curves) and the corresponding fits to Eq. 6 (solid curves).

The rotation of individual bacteria after switching the magnetic field was measured by bright-field time-lapse microscopy (Fig. 3a). For bacteria that were rotating mainly in the image plane, the angle *θ* of each bacterium relative to the magnetic field direction was tracked manually, and the resulting time course of the angle *θ*(*t*) (Fig. 3b) was fitted to the solution of the overdamped rotational equation of motion $\mu B\,\sin \,\theta ={\gamma }_{{\rm{rot}}}\frac{d}{dt}\theta$:

4 $${\rm{\theta }}(t)=2\,{\rm{arc}}\,\tan [{e}^{(-\frac{\mu \cdot B}{{\gamma }_{{\rm{rot}}}}\cdot t)}\cdot \,\tan (\frac{{{\rm{\theta }}}_{0}}{2})]$$

The parameter *γ*_rot_ is the rotational viscous drag coefficient of the bacterium, and *θ*_*0*_ is the angle of the bacterium at the beginning of the data acquisition at *t* = 0. The value for *γ*_rot_ was calculated for each individual bacterium from its shape parameters as described in the *Materials and Methods* section. The remaining free fit parameters were the magnetic moment *μ* and the value *θ*_*0*_. An overview over multiple time series of the rotation angle *θ* as a function of time *t* and the corresponding fits to Eq. 4 can be seen in Fig. 3c.

For bacteria that were mainly rotating perpendicular to the image plane, the time Δ*t* that was needed for a complete rotation was determined. According to Eq. 4, this time is

5 $${\rm{\Delta }}t=\frac{{\gamma }_{{\rm{rot}}}}{\mu \cdot B}\cdot \,\mathrm{ln}[\frac{{\tan ({\rm{\theta }}}_{e}/2)\,}{\tan ({{\rm{\theta }}}_{s}/2)}]$$

where θ_s_ is the angle of the bacterium at the start of the rotation, and θ_e_ is the angle of the bacterium at the end of the rotation (see also Penninga *et al*.^46^). The rotation time Δ*t* diverges for a rotation from 0° to 180°. The limited optical resolution^47^ leads to a limited precision in the determination of the orientation of a rotating object^32, 33, 48, 49^. We found that the tracking precision for the orientation of the bacteria was approximately 4°. Consequently orientation angles of up to 4° were indistinguishable from an angle of 0° and orientation angles of down to 176° were indistinguishable from an angle of 180°. We therefore used the boundary values of θ_s_ = 4° and θ_e_ = 176° for the calculation of ∆t. The value for *γ*_rot_ was also in this case determined for each individual bacterium from its shape parameters, as described in the *Materials and Methods* section and the resulting magnetic moment *µ* was calculated directly.

The rotation experiments were performed at magnetic field strengths that were sufficiently small to neglect the translational motion of the bacteria in the inhomogeneous field of the MT. The translational motion of the bacteria during their rotation was typically approximately 1 µm, which resulted in changes of the local magnetic fields of approximately 0.1 mT. These changes correspond to relative changes between 2% and 0.4% for the used magnetic fields between 6 mT and 23 mT, which was considered to be negligible. However, for sufficiently large magnetic fields, a strong translational motion of the bacteria along the gradient of the magnetic field was observed. These translational motions were used as a second method for the determination of the magnetic moment of the bacteria.

### Translation of bacteria

Bacteria with a magnetic moment ***µ*** experience a force ***F*** = ∇(***μ*** · ***B***) in a magnetic field ***B***. For a bacterium with a constant magnetic moment with the absolute value *µ*, which is aligned along a magnetic field with the magnitude *B*, the resulting overdamped translational equation of motion is $\nabla B={\gamma }_{{\rm{trans}}}\frac{d}{dt}r$, where *γ*_trans_ is the translational viscous drag coefficient and *r* is the distance of the bacterium to the MT tip. For the known magnetic field *B*(*r*) (Eq. 3) the solution of this equation is

6 $$r(t)={r}_{0{\rm{B}}}+{[{({r}_{0}-{r}_{0{\rm{B}}})}^{{c}_{{\rm{B}}}+2}-\mu {B}_{0}\frac{{d}_{0}^{{c}_{{\rm{B}}}}}{{\gamma }_{{\rm{trans}}}}{c}_{{\rm{B}}}({c}_{{\rm{B}}}+2)t]}^{\frac{1}{({c}_{{\rm{B}}}+2)}}$$

The parameter *r*_*0*_ is the distance of the bacterium to the MT tip at the beginning of the data acquisition at *t* = 0. This equation was fitted to the experimental data of the bacterial position as a function of time *r*(*t*), which was determined by manual tracking of the translational bacterial motion. The value for *γ*_trans_ was calculated for each individual bacterium from its shape parameters as described in the *Materials and Methods* section. For each bacterium, the remaining free fit parameters were its magnetic moment *µ* and the value *r*_0_. An overview of multiple time series of the displacement of the bacteria as a function of time and the corresponding fits to Eq. 6 is shown in Fig. 3d. The bacteria were tracked for several seconds, and the displacement during this time was on the order of up to ten micrometers (instead of absolute time- and space-axes, temporal and spatial scale bars were used in this figure to allow the representation of multiple displacement data sets).

These measurements were performed at magnetic fields ranging between 90 mT and 130 mT. The resulting magnetic moments of the bacteria were therefore induced magnetic moments that were expected to have values closer to the saturation moment than to the remanent moment^21^. The upper boundary of 130 mT was given by the maximal field strength that was created close to the MT tip, whereas the lower value of 90 mT was set by the boundary condition that a significant translational motion of the bacteria in the highly viscous fluid needed to be detectable within the measurement time.

### Viscous drag coefficients

The translational and rotational viscous drag coefficients were determined for each bacterium individually as described in the *Materials and Methods* section. To this end, we used two approaches that both take the helical shape and the dimensions of an individual bacterium into account. The end-to-end-length *L*_*ee*_ and the amplitude *A* (Fig. 1) were determined for each cell individually from light microscopy images. The diameter *d* of the bacterium is close to the resolution limit of diffraction-limited light microscopy^47^. We therefore analyzed the dimensions of 111 bacteria by TEM, and we found that the spread of the diameter values was very small. The average diameter was 420 nm, and the standard deviation was 30 nm; thus, we considered the mean diameter for the calculation of the drag coefficients of each bacterium. Similarly, the arc length *s* was difficult to determine by light microscopy. Consequently, we also determined this value from TEM micrographs from a sample of 125 bacteria. We found that the ratio of the arc length to the end-to-end-length was relatively well defined with a value of *s*/*L*_ee_ = 1.1 ± 0.1. Thus, we used this value to further calculate the arc length of each bacterium from its light-microscopically determined end-to-end-length: *s* = 1.1 *L*_ee_.

With the given dimensions for each bacterial cell, we determined the translational and rotational drag coefficients in a Stokes flow. Within the Stokesian dynamics, the surface of the bacteria was divided and represented by up to *N* = 10,000 particles interacting hydrodynamically via the Oseen tensor. The Boundary Integral Method uses a surface discretization of approximately 24,000 flat triangles and solves the Stokes equation by computing the surface velocities from a specified boundary traction.

**Figure 4:**
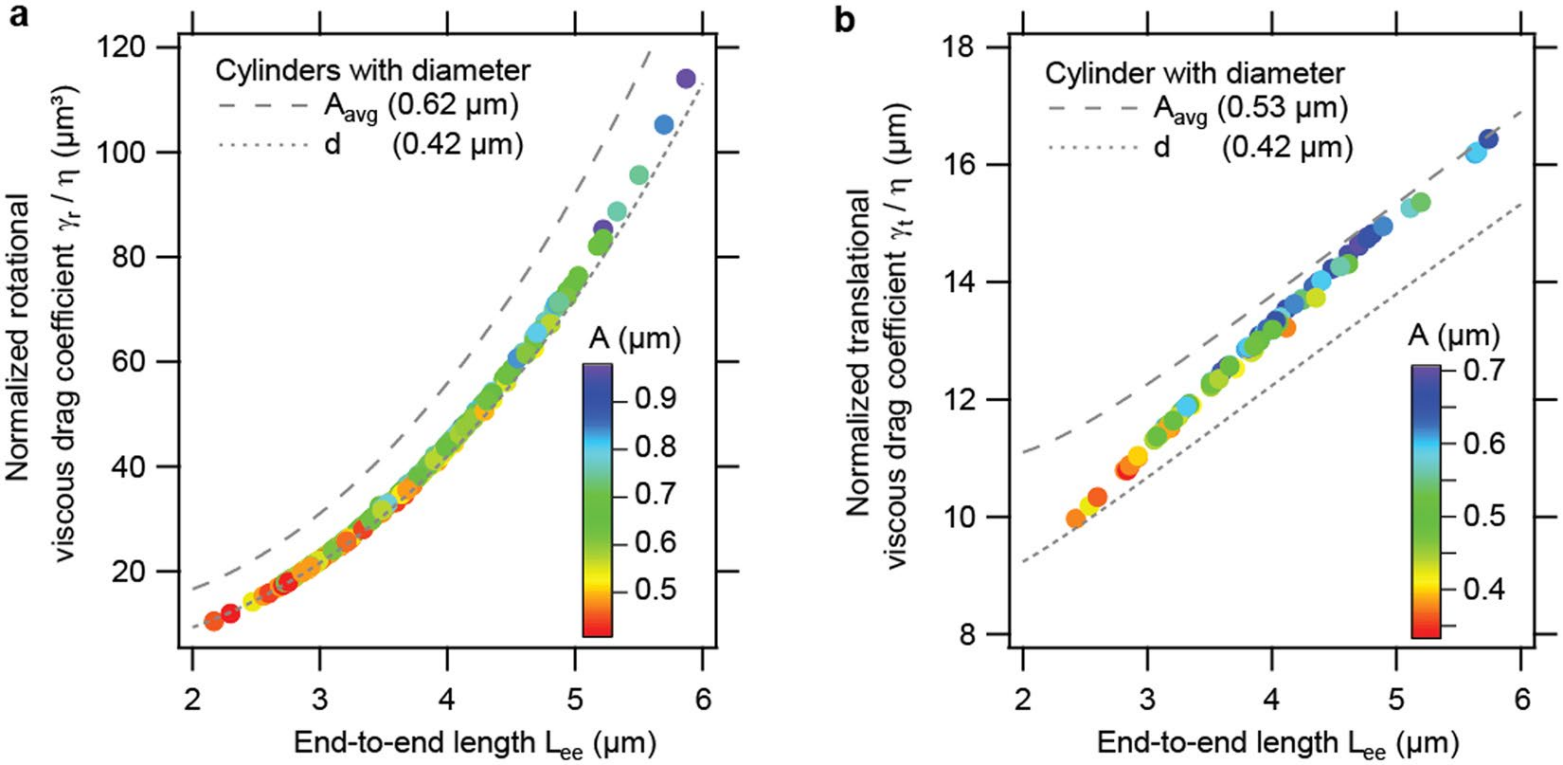
Normalized viscous drag coefficients of individual *M. gryphiswaldense* cells as a function of bacterial dimensions. The rotational (**a**) and translational (**b**) viscous drag coefficients were both calculated with Stokesian dynamics and with a Boundary Integral Method. Shown are the mean values of both methods, which deviate only negligibly from each other. The viscous drag coefficients are normalized by the viscosity of the medium *η* and plotted as a function of the end-to-end length *L*_ee_ of the bacteria. The amplitude *A* of the bacteria is indicated by the color of the data points. For comparison purposes, the normalized viscous drag coefficients of cylinders with various diameters are also shown.

The values of the viscous drag coefficients calculated by the two different methods provided quantitatively very similar results for each bacterium. For the rotational viscous drag coefficients, the two methods had an average discrepancy of 0.7% while the translational viscous drag coefficients differed by an average of 1.5%. We therefore used the mean value of the two methods as the drag coefficient for each bacterium. The calculated drag coefficients are shown in Fig. 4 as a function of the end-to-end length of the bacteria. The color code of the data points represents the amplitude *A* of the bacteria. Longer bacteria, i.e., bacteria with a larger end-to-end length *L*_ee_ tended to have a larger amplitude. The drag coefficients shown in Fig. 4 are normalized to the viscosity of the liquid *η*, which varied slightly from experiment to experiment because the temperature in the laboratory varied slightly in the range of 20.8 °C to 23.3 °C.

Both drag coefficients are larger than the drag coefficients of cylinders with a diameter that equals the diameter of the bacteria which had an average value 0.42 µm. For a long cylinder with a length *L*, a diameter *d* and *L* ≫ *d*/2, the normalized rotational viscous drag coefficient can be approximated^50^ by

7 $$\frac{{\gamma }_{{\rm{rot}}}}{\eta }=\frac{1/3\cdot \pi \cdot {L}^{3}}{\mathrm{ln}(\frac{L}{d})-0.66}$$

and the normalized translational viscous drag coefficient can be approximated by

8 $$\frac{{\gamma }_{{\rm{trans}}}}{\eta }=\frac{2\pi \cdot L}{\mathrm{ln}(\frac{L}{d})-0.20}$$

Furthermore, both drag coefficients are always smaller than the drag coefficients of cylinders with a diameter that corresponds to the average value of the amplitude of the bacteria. These average amplitudes were *A*_avg_ = 0.62 µm and *A*_avg_ = 0.53 µm for the bacteria that were investigated in the rotational and translational experiments, respectively.

### Magnetic moments

**Figure 5:**
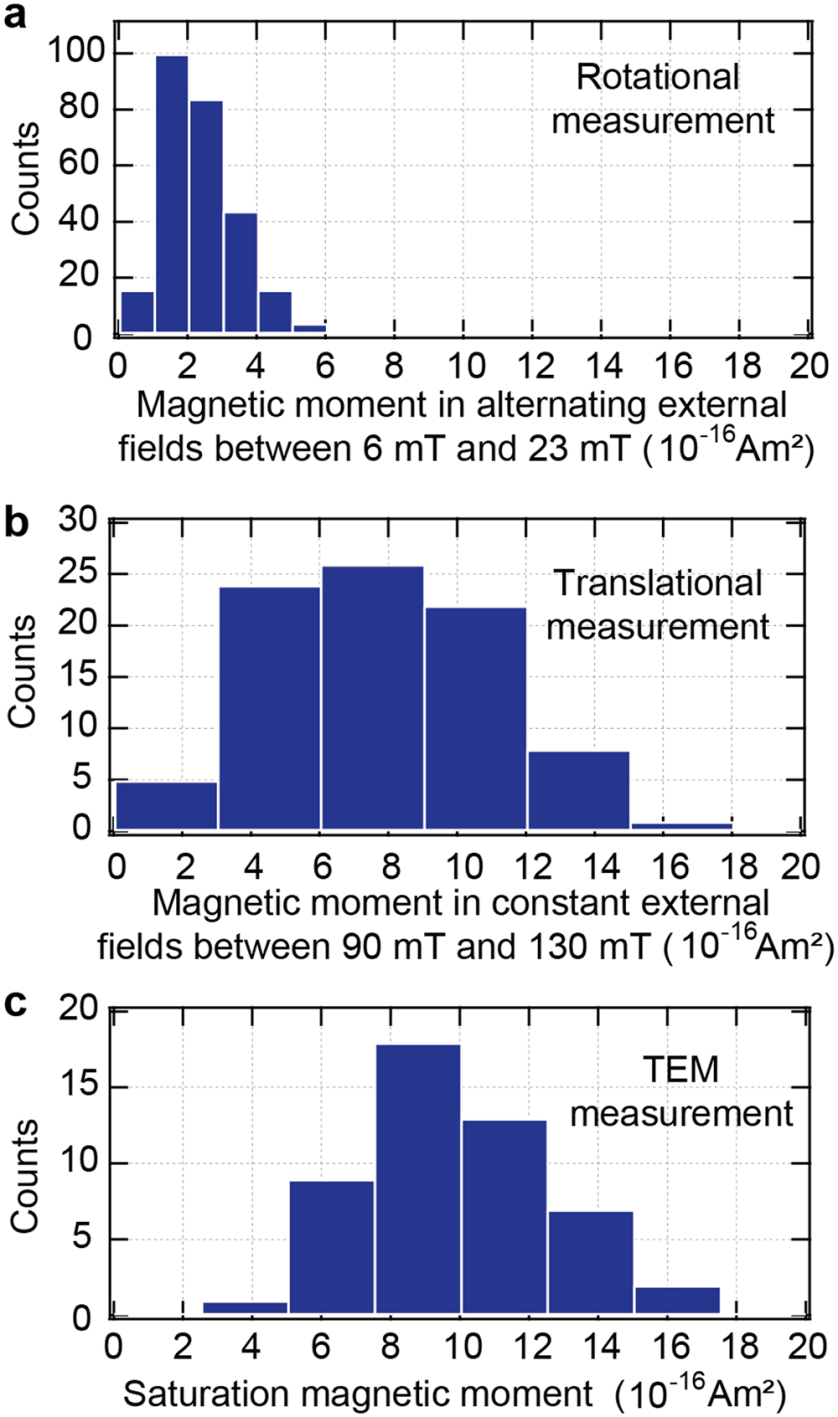
Distributions of the magnetic moments of single *M. gryphiswaldense* cells. (**a**) Magnetic moments determined by measuring the rotation of *N* = 265 cells in alternating magnetic fields. The field strengths ranged from B = 6 mT to 23 mT. (**b**) Magnetic moments determined by measuring the translation of *N* = 86 cells in static magnetic fields. The field strengths ranged from B = 90 mT to 130 mT. (**c**) Saturation magnetic moments of *N* = 50 cells determined by measuring the total magnetosome volume of each cell with TEM.

With the calculated rotational viscous drag coefficients, the magnetic moment of each bacterium was determined by fitting Eq. 4 to the tracked rotational motion for the case that the rotation was taking place mainly in the image plane. For the case that the rotation was mainly occurring perpendicular to the image plane, the magnetic moment of each bacterium was calculated by directly applying Eq. 5. As expected, the resulting distributions of magnetic moments were indistinguishable from each other, and we therefore pooled the data. We tracked the rotational motion of *N* = 265 bacteria and found an average magnetic moment of the cells of *μ* = 2.4 · 10^−16^ A m^2^ with a standard deviation of *σ*_*μ*_ = 1.1 · 10^−16^. The maximal magnetic moment was *μ*_max_ = 6.3 · 10^−16^ A m^2^, and the minimal moment was *μ*_min_ = 0.58 · 10^−16^ A m^2^. The magnetic field strengths for the rotational measurements were between *B* = 6 mT and *B* = 23 mT. The distribution of the magnetic moments from the rotational measurements is shown in Fig. 5a.

With the calculated translational viscous drag coefficients, the magnetic moment of each bacterium was determined by fitting Eq. 6 to the tracked translation motion. We tracked the translational motion of *N* = 86 bacteria and found an average magnetic moment of the cells of *μ* = 7.7 · 10^−16^ A m^2^ with a standard deviation of *σ*_*μ*_ = 3.4 · 10^−16^ A m^2^. The maximal magnetic moment was *μ*_*max*_ = 16 · 10^−16^ A m^2^, and the minimal moment was *μ*_min_ = 1.2 · 10^−16^ A m^2^. The magnetic field strengths for the translational measurements were between *B* = 90 mT and *B* = 130 mT. The distribution of the magnetic moments from the translational measurements is shown in Fig. 5b.

**Figure 6:**
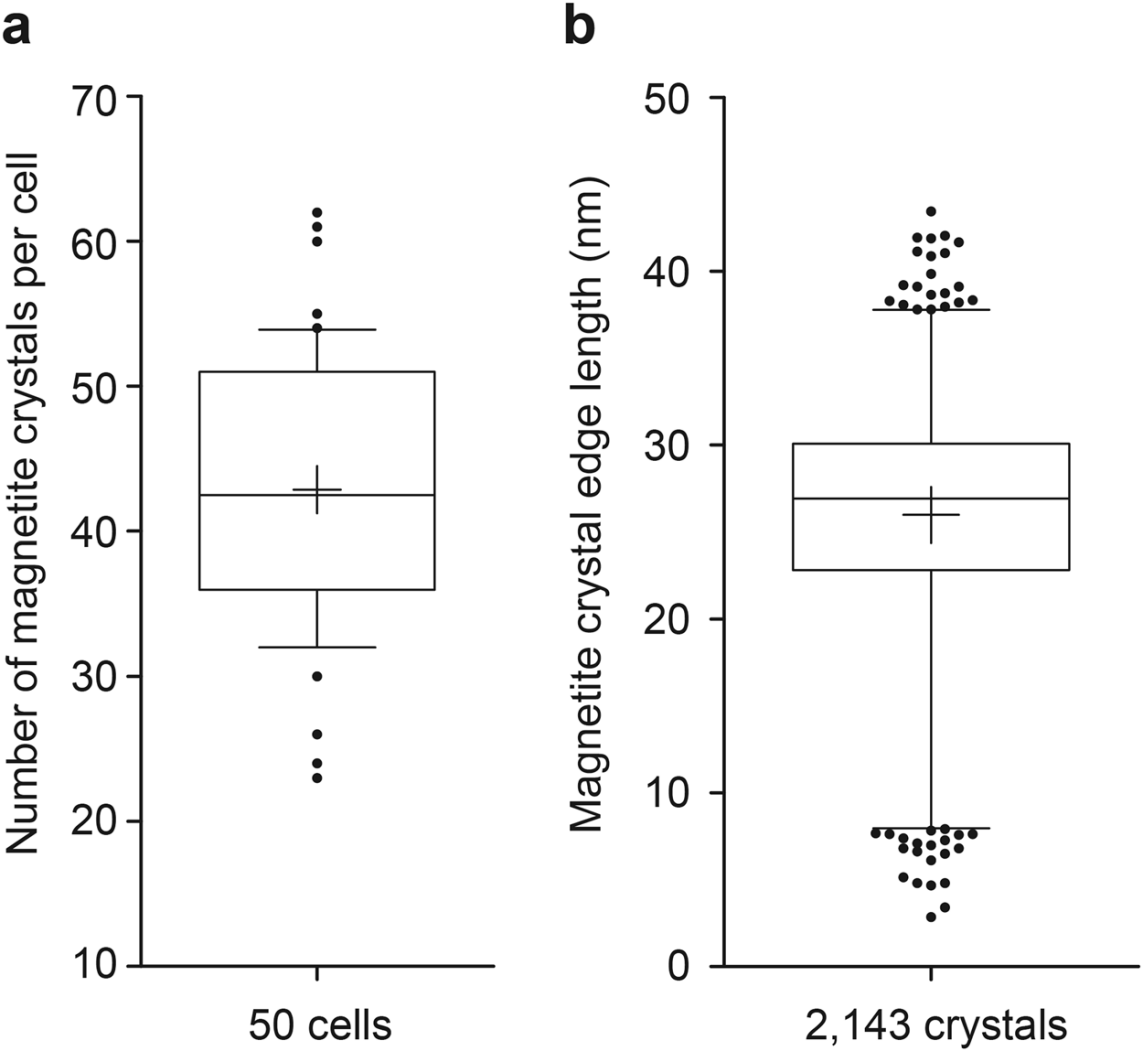
Distributions of the magnetite crystal numbers and sizes in *M. gryphiswaldense* cells. (**a**) Distribution of the number of magnetite crystals per cell. The data from 50 cells are represented in a box and whiskers plot. The box represents 50% of the central data, and the whiskers represent the 10–90 percentile. The central line depicts the median, and the cross indicates the average. (**b**) Distribution of the magnetite crystal edge length of 2,143 crystals from 50 cells. The box represents 50% of the central data and the whiskers show the 1–99 percentile. The central line depicts the median, and the cross indicates the average.

For a comparison, we determined the saturation magnetic moment for *N* = 50 bacteria by determining the total magnetosome volume *V*_mag_ for each cell by TEM. This value was multiplied by the saturation magnetization of magnetite^51^ $M=4.8\cdot {10}^{-22}\frac{{{\rm{A}}{\rm{m}}}^{2}}{{{\rm{n}}{\rm{m}}}^{3}}$ to determine the saturation magnetic moment for each cell *μ* = *V*_mag_ · *M*. The average number of magnetite crystals per magnetosome chain was 43 ± 10 (*N* = 50 cells) with chains harboring between 23 and 62 crystals (Fig. 6a). Overall we found magnetosomes with edge lengths between 3 nm and 43 nm with a mean of (26 ± 6) nm for *N* = 2,143 crystals (Fig. 6b). The average saturation magnetic moment of the cells was *μ*_sat_ = 9.9 · 10^−16^ Am^2^ and the standard deviation was ${\sigma }_{{\mu }_{{\rm{s}}{\rm{a}}{\rm{t}}}}=2.6\cdot {10}^{-16}{{\rm{A}}{\rm{m}}}^{2}$. The maximal saturation magnetic moment was *μ*_sat,max_ = 16.7 · 10^−16^ Am^2^ and the minimal moment was *μ*_sat,min_ = 4.9 · 10^−16^ Am^2^. The distribution of the saturation magnetic moments from the TEM measurements is shown in Fig. 5c. An overview of the magnetic moments determined by the three different methods (bacterial rotation, bacterial translation and magnetosome volume) is shown in Table 1.

**Table 1:** Magnetic moments of the bacteria measured by the three different methods and the corresponding external magnetic fields.

B (mT)	µ (10^−16^ A m^2^)	Method
6–23	2.4 ± 1.1	Bacterial rotation in alternating fields with a strength ranging between 6 mT and 23 mT
90–130	7.7 ± 3.4	Bacterial translation in constant fields with a strength ranging between 90 mT and 130 mT
∞	9.9 ± 2.6	Magnetosome volume determination by TEM

Average magnetic moments *μ* (±standard deviations) determined by the three different experimental methods: Measurement of the bacterial rotation in alternating external magnetic fields, measurement of the bacterial translation in constant external magnetic fields and determination of the magnetosome volume by TEM. The corresponding external field strengths *B* were in the range between 6 and 23 mT for the rotation measurements and between 90 and 130 mT for the translational measurements. The TEM measurement of the magnetosome volume provides the saturation moment for very large external magnetic fields.

## Discussion and Conclusions

We present a magnetic tweezers-based method for measuring the magnetic moments of individual bacteria, and we demonstrate the method by quantifying the individual magnetic moments of a large number of *M. gryphiswaldense* cells. Our method takes into account the helical shape of the bacteria and it can be adapted to allow the investigation of arbitrarily-shaped bacteria. Key parameters that describe the size and the shape of the bacteria, their end-to-end length and amplitude, were measured for each individual cell for the determination of its magnetic moment. Furthermore, our method is based on biologically inert (dead) yet well-preserved cells to avoid the non-thermal noise induced by living cells. In addition to characterizing the magnetic moment on a single cell level, our approach can also address various questions concerning the large spread of magnetic moments that were previously reported.

One of these questions was whether it is necessary to take the helical shape of *M. gryphiswaldense* into account or whether it is sufficient to approximate them with a simplified geometry, such as a cylinder. We found (Fig. 4) that for the rotational measurements, the measured end-to-end lengths of the bacteria varied from *L*_ee,min_ = 2.2 µm up to *L*_ee,max_ = 5.9 µm with a mean value of 〈*L*_ee_〉 = 3.7 ± 0.7 μm. A cylinder with a length of *L* = 3.7 µm and a diameter of *d* = 0.42 µm (which is the average diameter according to our TEM measurements) has a normalized rotational viscous drag coefficient of approximately $\frac{{\gamma }_{{\rm{rot}}}}{\eta }=35.2\,\mu {{\rm{m}}}^{3}$. Our exact calculations of the viscous drag, which take the helical shape and the individual dimensions of the cells into account, yield an average normalized rotational viscous drag coefficient of $\langle \frac{{\gamma }_{{\rm{rot}}}}{\eta }\rangle =38.7\pm 17.0\,\mu {{\rm{m}}}^{3}$. Therefore, calculating the average magnetic moment of the cells using the mean value 〈*L*_ee_〉 and approximating the cells as cylinders instead of taking the real helical cell shape into account, leads to an error of approximately 9%. Similarly for the translational measurements, the measured end-to-end lengths of the bacteria varied from *L*_ee, min_ = 2.4 µm up to L_ee,max_ = 5.7 µm with a mean value of 〈*L*_ee_〉 = 3.8 ± 0.7 μm. A cylinder with a length of *L* = 3.8 µm and a diameter of *d* = 0.42 µm has a normalized translational viscous drag coefficient of approximately $\frac{{\gamma }_{{\rm{trans}}}}{\eta }=11.9\,\mu {\rm{m}}$. Our exact calculations of the viscous drag, which take the helical shape and the individual dimensions of the cells into account yield an average normalized translational viscous drag coefficient of $\langle \frac{{\gamma }_{{\rm{trans}}}}{\eta }\rangle =12.8\pm 1.4\,\mu {\rm{m}}$. Therefore, calculating the average magnetic moment of the cells using the mean value 〈*L*_ee_〉 and approximating the cells as cylinders instead of taking their real helical shape into account, leads to an error of approximately 7%. From these observations, we can conclude that for the given bacterial population, the approximation of the cell shapes by a single cylinder with a length and a diameter given by the average length and diameter of the population is a reasonable approach if systematic errors on the order of magnitude of approximately 10% are acceptable.

Another raised question was whether it is necessary to take the dimension of each individual cell into account or whether it is sufficient to use average dimensions of a bacterial ensemble. If the study is purely focused on the average magnetic properties of a bacterial ensemble and if an uncertainty of 10% is acceptable, using the average dimensions is sufficient as stated above. However if individual magnetic moments of single bacteria are relevant, using the individual dimensions of the cells is necessary as discussed below. Figure 4 shows that the individual length and amplitude of the bacteria is important for determining the individual viscous drag and thus the magnetic moment of each bacterium. The rotational viscous drag varies by more than a factor of 10 between the smallest value of $\frac{{\gamma }_{{\rm{rot}}}}{\eta }=10.5\,{\mu m}^{3}$ for the shortest bacterium and the largest value of $\frac{{\gamma }_{{\rm{rot}}}}{\eta }=114\,{\mu m}^{3}$ for the longest bacterium. If the average rotational viscous drag value was used instead of the drag value based on the individual size of the cells, the magnetic moment of the shortest bacterium would be overestimated by +270% and the magnetic moment of the longest bacterium would be underestimated by −64%. In addition, Fig. 4 shows that the translational viscous drag varies between a value of $\frac{{\gamma }_{{\rm{trans}}}}{\eta }=9.96\,\mu {\rm{m}}$ for the shortest bacterium and a value of $\frac{{\gamma }_{{\rm{trans}}}}{\eta }=16.4\,\mu {\rm{m}}$ for the longest bacterium. Thus, if the average translational viscous drag value was used instead of the drag value based on the individual size of the cells, the magnetic moment of the shortest bacterium would be overestimated by +29% and the magnetic moment of the longest bacterium would be underestimated by −22%. The observation that the rotational measurement of the magnetic moment is more sensitive to the length of the bacteria than the translational measurement can be understood by the circumstance that the rotational viscous drag coefficient scales approximately with the cube of the bacteria’s length, whereas the translational drag coefficient scales approximately only linearly with the length of the bacteria.

The last question was whether the use of dead cells (ruling out the induction of non-thermal noise) leads to more consistent results than the use of living cells if different methods for determining the magnetic moments are applied. Since we only used dead cells in our measurements, we cannot provide a final answer to this question. However, we are able to contribute to an answer by testing whether the three different methods that were applied in our study are consistent with previous measurements on ensembles of non-motile bacteria. Fischer *et al*. measured the relative magnetization behavior of a large ensemble of dead *M. gryphiswaldense* cells^21^. These measurements provide information about the change of the average magnetic moment of the bacteria as a function of an external magnetic field. Their data indicate that the direction of the bacterial magnetization is reversed if external fields with an absolute value of more than approximately 20 mT are applied in the direction that is antiparallel to the magnetic fields of the bacteria. In agreement with this magnetic coercivity, we observed a rotation of the bacteria after a reversal of the magnetic field direction only for magnetic field strengths of less than 23 mT. For larger magnetic fields, the bacteria did not rotate suggesting a reversal of the direction of their magnetic moment relative to the orientation of the bacteria.

Furthermore we derived magnetic moments with a mean value of *μ* = 7.7 · 10^−16^ A m^2^ and a standard deviation of *σ* = 3.4 · 10^−16^ A m^2^ from our measurements of the bacterial translation in constant external fields (field strength: 90 mT–130 mT). Given the *N* = 86 translation measurements, the standard error of the mean is therefore $\frac{1}{\sqrt{86}}\cdot 3.4\cdot {10}^{-16}{{\rm{A}}{\rm{m}}}^{2}=0.4\,\cdot {10}^{-16}{{\rm{A}}{\rm{m}}}^{2}$. The TEM measurements of the saturation magnetic moment for *N* = 50 cells yielded a mean value of *μ* = 9.9 · 10^−16^ A m^2^, a standard deviation of *σ* = 2.6 · 10^−16^ A m^2^ and therefore a standard error of the mean of $\frac{1}{\sqrt{50}}\cdot 2.6\cdot {10}^{-16}{{\rm{A}}{\rm{m}}}^{2}=0.4\cdot {10}^{-16}{{\rm{A}}{\rm{m}}}^{2}$. A combination of these two measurements shows that in external magnetic fields in the range between 90 mT and 130 mT the cells have magnetic moments that correspond to a value of 78% **±** 5% of the saturation magnetic moment. For these field strengths, the measurements of Fischer *et al*.^21^ yielded magnetic moments that correspond to 95% **±** 3% of the saturation magnetic moments. Although these measurements are not completely in agreement, they agree relatively well compared to the large discrepancies on the order of one magnitude that were reported so far. Furthermore, our results are in agreement with the work of Reufer *et al*.^10^ who investigated *M. gryphiswaldense* and found an ensemble average of the magnetic moment of (2.0 ± 0.6) × 10^−16^ A m^2^ at a magnetic field of 1.5 mT. Based on the bacterial magnetization behavior^21^, an extrapolation of this magnetic moment from an external field of 1.5 mT to external fields in the range of 6 mT to 23 mT would result in a magnetic moment of (2.7 ± 1.2) × 10^−16^ A m^2^. This value is in very good agreement with the value of (2.4 ± 1.1) × 10^−16^ A m^2^ that we found for magnetic fields between 6 mT to 23 mT. Altogether, the agreement of our three methods with several previous measurements that were also based on dead cells indicates that the use of such cells leads to more consistent results than the use of living cells.

The possibility of measuring the magnetic moments of a large number of single bacteria by tracking their motion close to the tip of MT and by considering their individual shape and size allows addressing novel questions for the investigation of magnetotactic bacteria. In our study, we used MT in combination with bright-field microscopy. However MT can also be combined with other imaging modes such as fluorescence microscopy. Fluorescence labeling of the magnetosome chain to directly image the chain motion *in vivo* was recently established^7^ and can be used in future in combination with the single cell magnetic moment measurements presented here. Moreover, deleting genes that are involved in magnetosome biosynthesis can, for example, be used to investigate quantitatively the effects of these genes on the magnetic moments of the bacteria. Although our results indicate that the use of dead cells provides more robust results that the use of living cells, our MT-based methods can be extended to allow the use of living cells. In this case, our method can be used for example to correlate the magnetic moments of individual bacteria with their behavioral response to obtain a deeper understanding of magnetotaxis as a navigational mechanism.

## Electronic supplementary material

Supplementary Information
