# Supplementary material for: Measurement of the magnetic moment of single Magnetospirillum gryphiswaldense cells by magnetic tweezers
Source: Sci Rep. 2017 Jun 15;7:3558. doi: 10.1038/s41598-017-03756-z (PMC5472611; doi:10.1038/s41598-017-03756-z)
Supplement: Supplementary file 1 — Supplementary Information [file 41598_2017_3756_MOESM1_ESM.pdf]

SUPPLEMENTARY INFORMATION

Measurement of the magnetic moment of single *Magnetospirillum gryphiswaldense* cells by magnetic tweezers

C. Zahn[1], S. Keller[1], M. Toro-Nahuelpan[2,3], P. Dorscht[1], W. Gross[1], M. Laumann[4], S. Gekle[5], W. Zimmermann[4], D. Schüler[2], and H. Kress*[1]

[1]Biological Physics, Department of Physics, University of Bayreuth, Germany
[2]Department of Microbiology, University of Bayreuth, Germany
[3]Department of Molecular Structural Biology, Max Planck Institute of Biochemistry, Planegg-Martinsried, Germany.
[4]Theoretical Physics I, Department of Physics, University of Bayreuth, Germany
[5]Biofluid Simulation and Modeling, Department of Physics, University of Bayreuth, Germany
*corresponding author: holger.kress@uni-bayreuth.de

Materials and Methods

Calculation of the viscous drag coefficients via Stokesian dynamics

The centerline $\boldsymbol{r}_{\text{cl}}$ of the bacteria's helix with diameter A, length in axial direction L and helical pitch λ is given by

$$\boldsymbol{r}_{\text{cl}}(u) = \begin{pmatrix} \frac{A}{2}\cos(2\pi\, u) \\ \frac{A}{2}\sin(2\pi\, u) \\ \lambda\, u \end{pmatrix} \tag{1}$$

where u is varied between 0 and $\frac{L}{\lambda}$ to describe the complete helix centerline. Any position $\boldsymbol{r}_{\text{H}}$ on the bacteria's surface, other than the ends, can be described by

$$\boldsymbol{r}_{\text{H}}(u,v) = \boldsymbol{r}_{\text{cl}}(u) + d\, \boldsymbol{D}_{\hat{e}_{\text{t}}}(v)\hat{e}_{\text{n}} \tag{2}$$

since every position has the same distance d to the centerline (except the ends). The unit vector $\hat{e}_{\text{n}}$ is the vector normal to the centerline and $\boldsymbol{D}_{\hat{e}_{\text{t}}}(v)$ denotes the rotation matrix by an angle $v \in [0, 2\pi]$ about an axis in direction of the local unit tangent vector $\hat{e}_{\text{t}}$ of the helix centerline. Through variations in the parameters v and u the bacteria's surface is parameterized. The both ends of the bacteria can be represented, in a similar manner, by fixing $u = 0$ or $u = \frac{L}{\lambda}$ and by varying d and v.

The diameter A is measured directly in the experiment. The quantities L and λ are determined by the experimentally measured arc length s

$$s = \int_0^{L/\lambda} du \left|\frac{\partial r_{\text{cl}}(u)}{\partial u}\right| = \sqrt{(\pi\, A)^2 + \lambda^2)}\,(L/\lambda) \tag{3}$$

and the measured end-to-end distance $L_{\text{ee}} = \left|\boldsymbol{r}_{\text{cl}}\left(\frac{L}{\lambda}\right) - \boldsymbol{r}_{\text{cl}}(0)\right|$.

The surface of the helix is divided and represented by N point-like beads with an identical effective radius a. They are located at positions $\boldsymbol{r}_k$ with $= 1, \ldots, N$. A force $\boldsymbol{F}_k$ is required to move the point-like particle at a position $\boldsymbol{r}_k$ through the fluid because the beads have a drag coefficient $\zeta = 6\pi\, \eta\, a$ in a solvent of viscosity η. The beads generate a flow field by moving through the fluid. The flow $\boldsymbol{u}_k(\boldsymbol{r})$ induced by the particle located at $\boldsymbol{r}_k$ at an arbitrary point in space $\boldsymbol{r}$ is given by

$$\boldsymbol{u}_k(\boldsymbol{r}) = \boldsymbol{O}(\boldsymbol{r} - \boldsymbol{r}_k)\boldsymbol{F}_k \tag{4}$$

with the Oseen tensor[1]

$$O_{ij}(\boldsymbol{r} - \boldsymbol{r}_k) = \frac{1}{8\pi\, \eta} G_{ij}(\boldsymbol{r} - \boldsymbol{r}_k)\,. \tag{5}$$

and the tensorial free space Greens function $G_{ij}\,(\boldsymbol{r}) = \frac{1}{r}\left(1 + \frac{r_i\, r_j}{r^2}\right)$. The parameters $i = 1,..3$ and $j = 1,..,3$ denote the entries of the matrices $\boldsymbol{O}$ and $\boldsymbol{G}$.

The flow field $\boldsymbol{u}_k(\boldsymbol{r})$, which is caused by particle k influences via the fluid, respectively via the Oseen tensor, the motion of all other particles at $\boldsymbol{r}_j$ with $j = 1,..,N$ and $j \neq k$. For a helix moving with the velocity $\boldsymbol{v}$, all N beads fixed on its surface move with the same speed $\dot{\mathbf{r}}_k = \boldsymbol{v}$ $(k = 1, \ldots, N)$. Every moving particle influences via the hydrodynamic interaction all other particles. Therefore, the forces $\boldsymbol{F}_1, \ldots, \boldsymbol{F}_N$ required to move the beads with a given velocity $\boldsymbol{v}$ are determined by the N coupled linear equations[1]

$$\boldsymbol{v} = \frac{\boldsymbol{F}_i}{\zeta} + \sum_{k=1,\neq i}^{N} \boldsymbol{O}(\boldsymbol{r}_i - \boldsymbol{r}_k)\, \boldsymbol{F}_k. \qquad (6)$$

For one solution with the velocity $\boldsymbol{v}$ nearly parallel to the helical axis and with the total force acting on the helical bacterium, $\boldsymbol{F} = \sum_k \boldsymbol{F}_k$ is parallel to $\boldsymbol{v}$. The friction coefficient γ_{trans} describes the proportionality between both quantities:

$$\gamma_{\mathrm{trans}}\, \boldsymbol{v} = \boldsymbol{F}. \qquad (7)$$

For a helix rotating with a frequency ω around the axis of $\boldsymbol{\omega}$ through its center $\boldsymbol{r}_\mathbf{c} = \frac{1}{N}\sum_k \boldsymbol{r}_k$, the velocity of each bead is given by

$$\dot{\boldsymbol{r}}_k = (\boldsymbol{r}_k - \boldsymbol{r}_\mathrm{c}) \times \boldsymbol{\omega}. \qquad (8)$$

Eq. 6 can be used to determine the relation between the bead velocities and the required forces. The torque $\boldsymbol{M}$ acting on the helix can be expressed in terms of the forces: $\boldsymbol{M} = \sum_k (\boldsymbol{r}_k - \boldsymbol{r}_\mathrm{c}) \times \boldsymbol{F}_k$. For the case in which the rotational axis is nearly perpendicular to the helix axis, the two vectors $\boldsymbol{\omega}$ and $\boldsymbol{M}$ are related via the rotational friction: $\gamma_{\mathrm{rot}}\, \boldsymbol{\omega} = \boldsymbol{M}$.

Calculation of the viscous drag coefficients by Boundary Integral Method

The Boundary Integral Method (BIM) exploits the fact that the Stokes equation is linear and can therefore be rewritten as an integral equation[2] for the flow velocity $\boldsymbol{u}(\boldsymbol{r})$ at an arbitrary point $\boldsymbol{r}$ inside the infinite and initially quiescent fluid:

$$u_i(\boldsymbol{r}) = \frac{1}{4\pi\,\eta} \oint_S G_{ij}(\boldsymbol{r} - \boldsymbol{y})\, f_j(\boldsymbol{y})\, d\boldsymbol{y}, \qquad (9)$$

where summation over the repeated index j is implied, $\boldsymbol{y}$ is a point on the surface S of the bacterium, η is the fluid viscosity and $\boldsymbol{f}$ is the surface traction.

When the observation point $\boldsymbol{r}$ is moved to the surface, Eq. 9 can be converted to a linear system of equations[2] which in our implementation is solved by GMRES[3,4]. The surface integral in Eq. 9 is computed by discretizing the bacterial surface using flat triangles to interpolate between the surface nodes. To allow a direct comparison, the nodes of the triangles are taken at the same positions as in the Stokesian dynamics calculations (see above).

To impose a rotation/translation on the bacterium, each node is coupled by a harmonic spring to an auxiliary anchor point. During the simulation, these imaginary anchor points are translated/rotated with a prescribed (angular) velocity. By distributing the force of these springs over the local area surrounding each node, the force is converted into a surface traction $\boldsymbol{f}$, which is a term in Eq. 9. The solution of the linear system resulting from Eq. 9 then yields the surface velocity $\boldsymbol{u}$ from which the desired drag coefficients γ_{rot} and γ_{trans} and can be directly obtained. The translational/rotational velocities have been chosen to be small enough that the relation between force and velocity remains linear.

References

1 Leal, L. G. *Advanced Transport Phenomena.* (Cambridge University Press, 2007).

2 Pozrikidis, C. *Boundary integral and singularity methods for linearized viscous flow.* (Cambridge University Press, 1992).

3 Saad, Y. & Schultz, M. H. GMRES: A generalized minimal residual algorithm for solving nonsymmetric linear systems. *SIAM J. Sci. Comput.* **7**, 856-869, doi:10.1137/0907058 (1986).

4 Daddi-Moussa-Ider, A., Guckenberger, A. & Gekle, S. Long-lived anomalous thermal diffusion induced by elastic cell membranes on nearby particles. *Phys. Rev. E* **93**, doi:10.1103/PhysRevE.93.012612 (2016).
